# Supplementary material for: Expanding Broad Molecular Reflex Testing in Non-Small Cell Lung Cancer to Squamous Histology
Source: Cancers (Basel). 2024 Feb 23;16(5):903. doi: 10.3390/cancers16050903 (PMC10931067; doi:10.3390/cancers16050903)
Supplement: Supplementary file 1 [file cancers-16-00903-s001.zip › TableS1.pdf]

Supplementary Table S1: DNA- and RNA-based NGS results.

**Testing characteristics**	
NGS performed and evaluable	n=316
Marked tumor area examined	77 (24.4%)
Whole tissue area examined	239 (75.6%)
Tumor cell content (%) - median (range)	40 (10-90)
**Detected alterations**	
AKT1 mutation	2 (0.6%)
ALK mutation	4 (1.3%)
ALK fusion	4 (1.3%)
BRAF mutation	6 (1.9%)
CTNNB1 mutation	2 (0.6%)
DDR2 mutation	20 (6.3%)
EGFR mutation	7 (2.2%)
EGFR amplification	4 (1.3%)
EGFR fusion	2 (0.6%)
ERBB2 mutation	1 (0.3%)
ERBB2 amplification	2 (0.6%)
ERBB4 mutation	6 (1.9%)
FBXW7 mutation	10 (3.2%)
FGFR1 mutation	1 (0.3%)
FGFR1 amplification	6 (1.9%)
FGFR1 fusion	1 (0.3%)
FGFR2 mutation	3 (0.9%)
FGFR3 mutation	13 (4.1%)
FGFR3 amplification	1 (0.3%)
FGFR3 fusion	1 (0.3%)
KRAS mutation	15 (4.7%)
KRAS amplification	2 (0.6%)
MAP2K1 mutation	5 (1.6%)
MET mutation	14 (4.4%)
MET amplification	1 (0.3%)
MET Exon 14 skipping	1 (0.3%)
NOTCH1 mutation	2 (0.6%)
NRAS mutation	3 (0.9%)
PIK3CA mutation	37 (11.7%)
PIK3CA amplification	7 (2.2%)
PTEN mutation	20 (6.3%)
SMAD4 mutation	14 (4.4%)
STK11 mutation	5 (1.6%)
TP53 mutation	228 (72.2%)
