# Supplementary material for: Expanding Broad Molecular Reflex Testing in Non-Small Cell Lung Cancer to Squamous Histology
Source: Cancers (Basel). 2024 Feb 23;16(5):903. doi: 10.3390/cancers16050903 (PMC10931067; doi:10.3390/cancers16050903)
Supplement: Supplementary file 1 [file cancers-16-00903-s001.zip › TableS3.pdf]

Case	Molecular target	Co-Mutations	Targeted therapy	Treatment response
1	EML4-ALK fusion	no	no (early stage)	NA
2	EML4-ALK fusion	no	Alectinib	partial response
3	KRT6A-ALK fusion	no	Alectinib, Brigatinib	discontinued (adverse events)
4	SLC24A3-ALK fusion	no	no (early stage)	NA
5	EGFR-NUP160 fusion	TP53 p.H179R	no (early stage)	NA
6	MAD1L1-EGFR fusion	no	no (early stage)	NA
7	EGFR p.S768_D770dup	TP53 p.E298X	no (died before)	NA
8	EGFR p.L747_P753delinsS	no	Osimertinib	complete response
9	EGFR p.746_750del	TP53 p.177_182del	Osimertinib	partial response
10	EGFR p.L747_P753delinsS	TP53 p.M246fs	Osimertinib	partial response
11	EGFR p.L858R	SMAD4 p.W524X	Osimertinib (adjuvant)	relapse-free
12	EGFR p.L858R<br>EGFR p.S768I	TP53 p.R213Q	Osimertinib (adjuvant)	relapse-free
13	BRAF p.K601E	CDKN2A p.25_31del<br>STK11 p.A398V	NA	NA
14	KRAS p.G12C	TP53 p.V157F	NA	NA
15	KRAS p.G12C	PIK3CA p.H1047R<br>TP53 V225fs	no (before drug approval)	NA
16	KRAS p.G12C	TP53 p.V274F	NA	NA
17	KRAS p.G12C	TP53 p.R213L	no (early stage)	NA
18	KRAS p.G12C	no	no (before drug approval)	NA
19	KRAS p.G12C	TP53 p.P190fs<br>KEAP1 p.Q217X	no (died before)	NA
20	KRAS p.G12C	no	no (died before)	NA
21	METex14 skipping	TP53 p.P190L<br>MET p.T1010I	Capmatinib	progressive disease
