# Supplementary figures and images for: Expanding Broad Molecular Reflex Testing in Non-Small Cell Lung Cancer to Squamous Histology
Source: Cancers (Basel). 2024 Feb 23;16(5):903. doi: 10.3390/cancers16050903 (PMC10931067; doi:10.3390/cancers16050903)

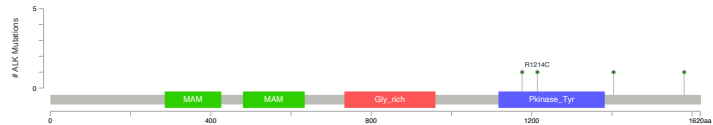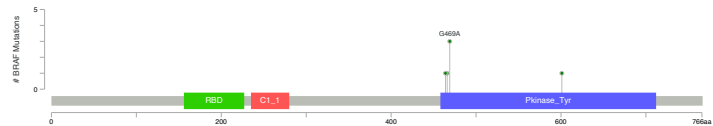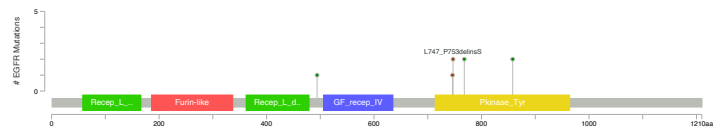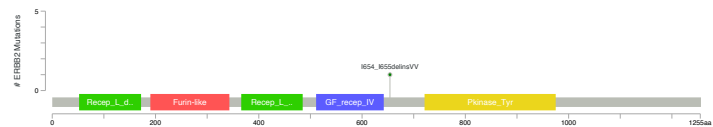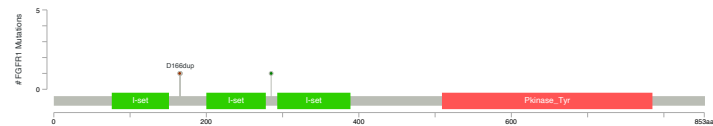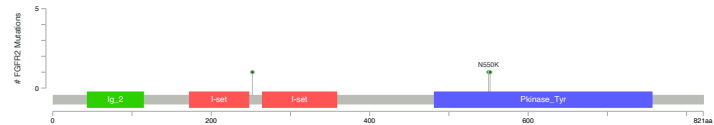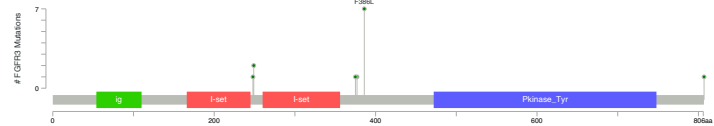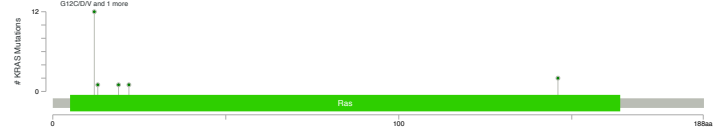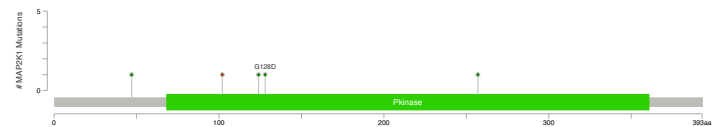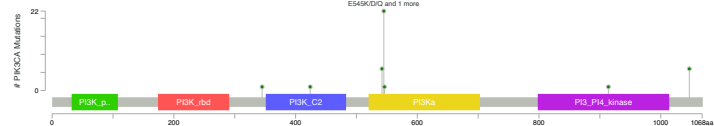

Supplement: Supplementary file 1 [file cancers-16-00903-s001.zip › FigureS1.pdf]

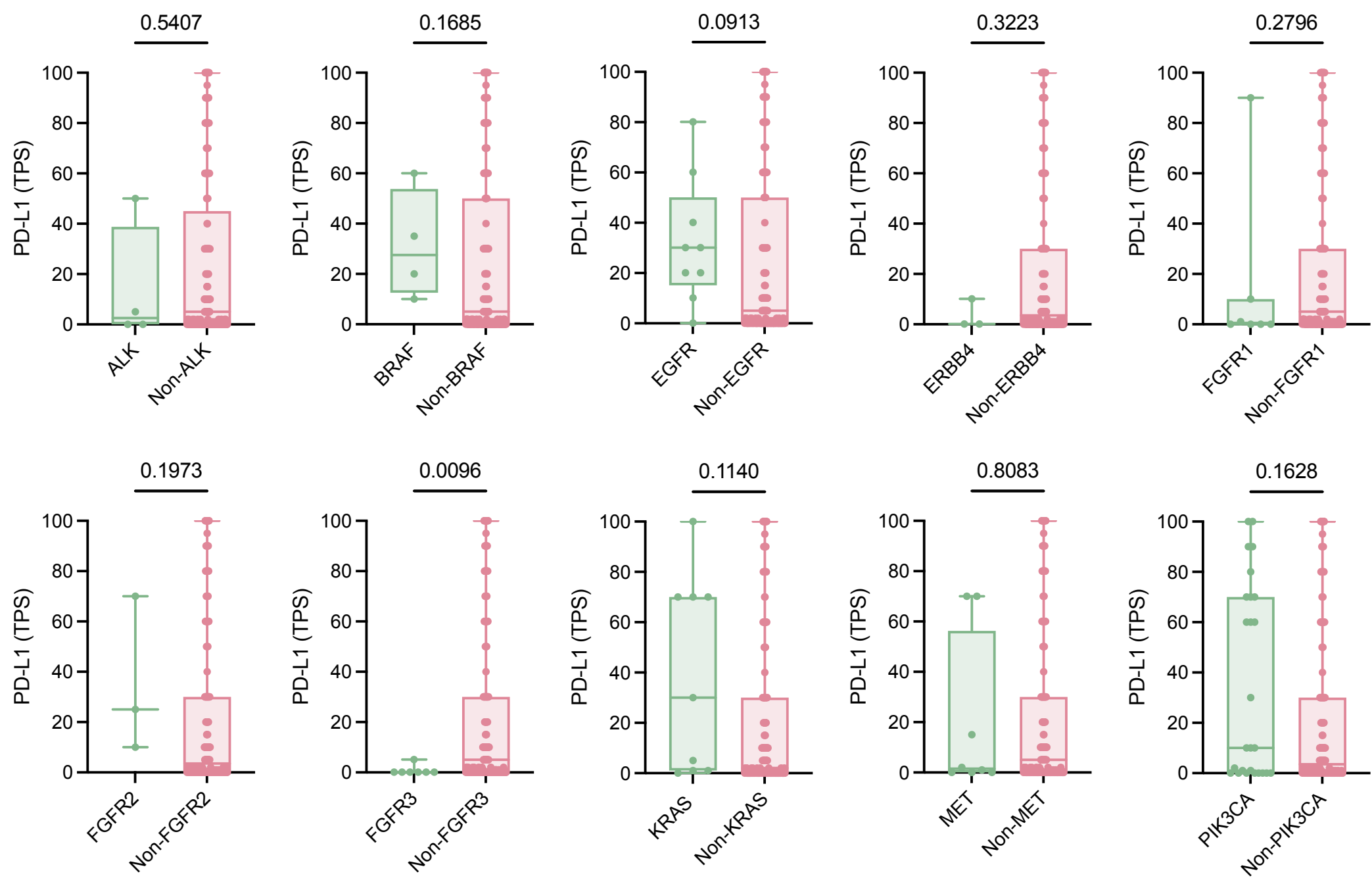

Supplement: Supplementary file 1 [file cancers-16-00903-s001.zip › FigureS2.pdf]

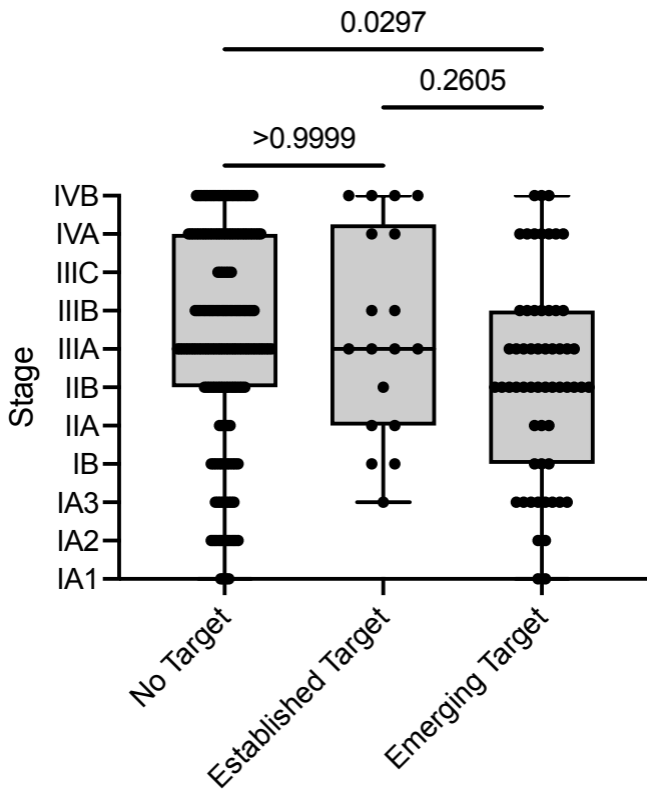

Supplement: Supplementary file 1 [file cancers-16-00903-s001.zip › FigureS3.pdf]
